# Perceptually motivated loss functions for computer generated holographic displays

**DOI:** 10.1038/s41598-022-11373-8

**Published:** 2022-05-11

**Authors:** Fan Yang, Andrew Kadis, Ralf Mouthaan, Benjamin Wetherfield, Andrzej Kaczorowski, Timothy D. Wilkinson

**Affiliations:** 1 Centre of Molecular Materials, Photonics and Electronics, University of Cambridge, Cambridge, UK; 2 Research Division, VividQ Ltd., Cambridge, UK

**Keywords:** Displays, Imaging and sensing

## Abstract

Understanding and improving the perceived quality of reconstructed images is key to developing computer-generated holography algorithms for high-fidelity holographic displays. However, current algorithms are typically optimized using mean squared error, which is widely criticized for its poor correlation with perceptual quality. In our work, we present a comprehensive analysis of employing contemporary image quality metrics (IQM) as loss functions in the hologram optimization process. Extensive objective and subjective assessment of experimentally reconstructed images reveal the relative performance of IQM losses for hologram optimization. Our results reveal that the perceived image quality improves considerably when the appropriate IQM loss function is used, highlighting the value of developing perceptually-motivated loss functions for hologram optimization.

## Introduction

Holography offers a unique ability to control light, which profoundly impacts various applications from optical telecommunications^1^, data storage^2^, microscopy^3^ to two- and three-dimensional displays^4,5^. Advances in algorithms and computational capacity have enabled Computer-Generated Holograms (CGHs) to be numerically calculated by simulating light diffraction and interference. The obtained CGH is displayed on a spatial light modulator (SLM), which modulates coherent illumination to reproduce the desired scenes. The goal of CGH algorithms is to compute a hologram that can be displayed on an SLM and that produces an intensity distribution that best approximates the desired image.

CGHs are commonly displayed on nematic liquid crystal SLMs, which boost superior light efficiency but are restricted to modulating only the phase of the incident beam. To solve the phase-only restriction imposed by these SLMs, double phase^4,6^ and error diffusion methods^7–9^ directly encode complex-amplitude diffraction fields into phase-only holograms. Another approach, known as the one-step phase retrieval algorithm (OSPR)^10,11^, displays multiple phase-only holograms within a short time interval to statistically average out errors in the replay field. Trained deep learning-based CGH algorithms are also employed as non-iterative solutions^12–14^. Iterative CGH algorithms such as direct search (DS)^15^ and simulated annealing (SA)^16^ alter single pixels in the hologram to find the optimal hologram. Phase retrieval methods like the Gerchberg-Saxton algorithm (GS)^17^ and hybrid input–output (HIO)^18,19^ method have also been explored.

Recently, the gradient descent method has been applied to phase-only CGH optimization^12–14,20–24^. The gradient of a predefined objective function is calculated and used to update the hologram phase at each iteration. This method can be further combined with a camera as a feedback optimization strategy to eliminate optical artifacts in experimental setups^13,22^. The specific loss function selected is essential in these iterative optimization approaches to drive the hologram phase to its optimal state. A standard choice of the loss function is the mean squared error (MSE) due to its simplicity of use and clear physical meaning. Though MSE quantifies the per-pixel error in the reconstructed image, it is widely criticized for its poor correlation with perceptual quality^25–28^.

A promising but relatively less exploited approach is to use image quality metrics (IQMs) in the phase-only CGH optimization process. The traditional role of IQMs in digital holography is to dynamically monitor the optimization process and to evaluate the perceptual quality of obtained images^29–32^. Modern IQMs model assesses visual quality based on a priori knowledge regarding the human visual system or uses learned models trained with large datasets. They use image features in appropriate perceptual spaces^28,33^ for image quality evaluation but have not yet been fully exploited in the CGH optimization process. Here, we focus on the use of IQMs as an alternative to the ubiquitous MSE for the training loss, with the intention of using the gradient of these perceptual metrics to strive for a better CGH optimization algorithm. The use of perceptual motivated loss functions has recently gained attention in foveated CGH^34,35^, focusing specifically on speckle suppression in the foveal region and peripheral perception. Other non-holographic image restoration applications have also explored perceptual losses, though it is observed that there is no single loss function that outperforms all others across different applications^36–38^.

In this paper, we present a comprehensive comparison of different IQMs as losses for CGH optimization using gradient descent. Specifically, we first choose ten optimization-suitable IQMs together with mean absolute error (MAE) and MSE to generate CGHs. These IQMs have not been applied to the hologram design, and are selected among the plethora of existing metrics due to their well establishment as well as their differentiability, a requirement for use in the gradient descent method. We build a holographic display prototype to acquire an optical reconstruction dataset of IQM optimization phase holograms. We use this dataset to perform an in-depth analysis of the relative performance of IQM losses based on extensive objective quality assessments as well as subjective comparisons informed by human perceptual judgments. Finally, we present a rigorous procedure for evaluating the perceptual quality of holographic images and highlight the value of developing perceptually-motivated loss functions for hologram optimization.

## Background

### CGH optimization model using the gradient descent method

**Figure 1:**
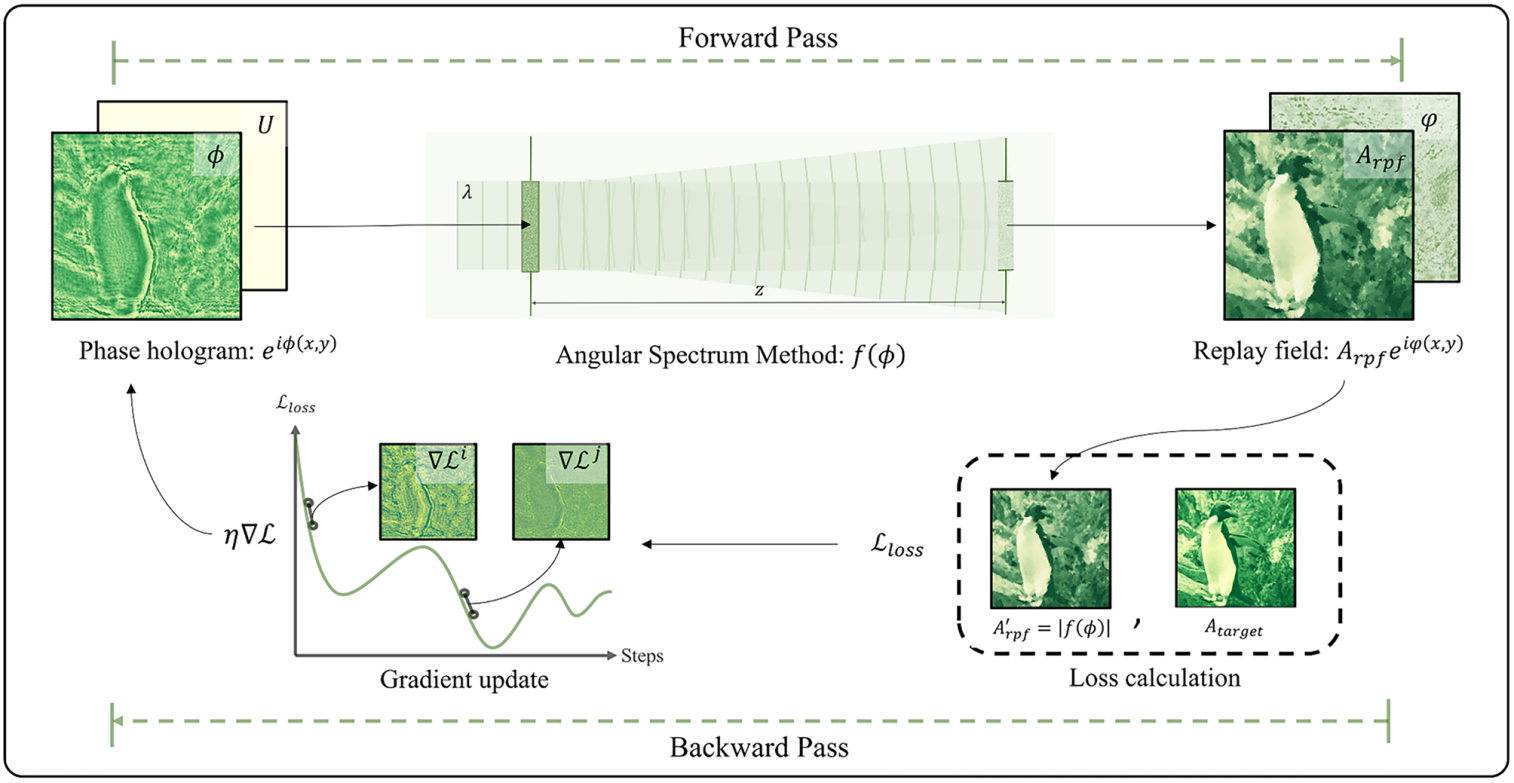
CGH optimization model based on the gradient descent method.

CGH generation based on the gradient descent method can be generalized as an optimization model. In the forward pass, the model propagates a phase hologram to the replay plane to produce a reconstructed image, which is used to calculate the loss by comparing it to the target image. In the backward pass, the model traverses backward from the output, collecting the derivatives of the loss function with respect to the phase hologram and updating the hologram to minimize the loss. The model iteratively goes through the forward pass and the backward pass to obtain the optimized phase hologram. This process is illustrated in Fig. 1.

In the forward pass, we consider the angular spectrum method^39,40^ with planar illuminating wave for modeling the diffraction propagation function:

1 $$f\left(\phi \right)={\mathcal{F}}^{-1}\left\{\mathcal{F}\left\{{e}^{i\phi \left(x,y\right)}\right\}\times exp\left[j2\pi z\sqrt{\frac{1}{{\lambda }^{2}}-{f}_{x}^{2}-{f}_{y}^{2}}\right]\right\}.$$

Here, $\phi \left(x,y\right)$ is the phase hologram that has been quantized so that it can be displayed on a binary or 8-bit SLM, $\lambda$ is the wavelength, ${f}_{x} , {f}_{y}$ are spatial frequencies, and $z$ is the propagating distance between the hologram plane and the replay field plane. $\mathcal{F}$ and ${\mathcal{F}}^{-1}$ denote the Fourier transform and the inverse Fourier transform, respectively. The resulting field $f\left(\phi \right)$ is a complex replay field, whose amplitude is related to the reconstructed image intensity by $I\left(\mu ,\nu \right)= {\left|f\left(\phi \right)\right|}^{2}$. To evaluate the perceived image quality, the amplitude of the replay field ${A}_{rpf}$ is compared with the target amplitude ${A}_{target}$ using a loss function $\mathcal{L}$. Though intensity-based objective functions can also be utilized for image quality evaluation, amplitude-based objective functions have been found to yield better algorithmic performance and are preferable in hologram optimization^41,42^. Therefore, the CGH optimization algorithm aims to find the optimal quantized phase hologram $\widehat{\phi }$ that minimizes the loss function $\mathcal{L}$ describing the visual quality, calculated from the reconstructed image amplitude $\left|f\left(\phi \right)\right|$ and the intended target image amplitude ${A}_{target}$:

2 $$\widehat{\phi } =\underset{\phi }{\mathit{argmin}}\mathcal{L}\left(s\cdot \left|f\left(\phi \right)\right|,{A}_{target}\right),$$

where $s$ is a scaling factor for normalization. The mean square error (MSE) for a $m$ by $n$ sampling points is commonly used as the loss function, computed by averaging the squared amplitude differences of reconstructed and target image pixels:

3 $${\mathcal{L}}_{MSE}=\frac{1}{mn}\sum_{m,n}{\left[\left|f\left(\phi \right)\right|-{A}_{target}\right]}^{2}.$$

In the backward pass, the model calculates the gradient $\partial \mathcal{L}/\partial {\phi }^{k-1}$ of the loss function with respect to the current estimate of the phase hologram ${\phi }^{k-1}$ to update the next estimate phase ${\phi }^{k}$. The gradient can be calculated by the chain rule, which involves the calculation of complex derivatives:

4 $$\frac{\partial \mathcal{L}}{\partial {\upphi }^{\mathrm{k}-1}}=\frac{\partial \mathcal{L}}{\partial {\mathrm{A}}_{\mathrm{rpf}}^{\mathrm{^{\prime}}}}\cdot \frac{\partial {\mathrm{A}}_{\mathrm{rpf}}^{\mathrm{^{\prime}}}}{\partial \mathrm{f}}\cdot \frac{\partial \mathrm{f}}{\partial {\upphi }^{\mathrm{k}-1}},\mathrm{ f}: {\mathbb{C}}\to {\mathbb{C}}.$$

In complex analysis, the holomorphic requirement for functions to be complex differentiable is very strict. Wirtinger calculus relaxes this requirement and allows approximate complex derivatives of nonholomorphic functions to be more easily calculated by using a conjugate coordinate system^21,43,44^. Recently, Wirtinger calculus has been implemented in automatic differentiation packages in machine learning libraries such as TensorFlow and PyTorch. These automatic differentiation packages keep a record of all the data and operations that have been done in the forward pass in a direct acyclic graph and automatically compute gradients using the chain rule. For a learning rate $\eta$, the next estimate phase hologram ${\phi }^{\left(k\right)}$ is given by:

5 $${\phi }^{\left(k\right)}={\phi }^{\left(k-1\right)}-\eta \nabla \mathcal{L}\left(s\cdot \left|f\left({\phi }^{\left(k-1\right)}\right)\right|,{A}_{target}\right).$$

Several update strategies, such as Adagrad^45^ and Adam^46^, propose learning rate update rules to improve accuracy and convergence speed.

### IQM as loss functions

IQMs play a vital role in the development and optimization of image processing and restoration algorithms. Generally, IQMs can be classified into full-reference methods, reduced-reference methods, and no-reference methods according to the availability of the original reference image. Since the target image is available in the CGH optimization model, we only consider full-reference methods as loss functions. IQMs are a function of a number of parameters, and different IQM implementations can yield significantly different results, impacting the performance of CGH optimization. We therefore consider ten differentiable full-reference IQMs from existing libraries IQA^37^ and PIQ^47^, benchmarked on common databases, which we believe include a wide range of state-of-art full-reference IQMs. We also include MAE and MSE as standards for comparison. Therefore, this IQM collection includes three error visibility methods: MSE, MAE and NLPD^33^, six structural similarity methods: SSIM^26^, MS-SSIM^48^, FSIM^49^, MS-GMSD^50^, VSI^51^, HaarPSI^52^, one information-theoretical method: VIF^53^, and two learning-based methods: LPIPS^25^ and DISTS^54^. Error visibility methods calculate the image error on a pixel-by-pixel basis. Structural similarity methods consider the perceived variation, including luminance, contrast, and structure, to assess image distortion. Information-theoretic methods quantify the amount of information loss in the distorted images with respect to the target images. Learning-based methods propose neural networks trained with numerous pictures to assess image quality. Table 1 summarizes the library of the IQMs considered as well as the underlying principle. The IQM is reformulated where necessary so that a lower score indicates higher predicted quality. For example, if the selected IQM is $SSIM$, then $\mathcal{L}$ is rewritten as ${\mathcal{L}}_{SSIM}= 1 - SSIM$.

**Table 1:** The utilized underlying principle of IQM losses for CGH optimization.

IQM losses	Library	Underlying principle
MAE	Pytorch	Pixel-based absolute error with average pooling
MSE	Pytorch	Pixel-based squared error with average pooling
NLPD	IQA	Root MSE in the weighted Laplacian pyramid decomposition domain
SSIM	IQA	A weighted combination of measures: luminance, contrast and structure
MS-SSIM	IQA	The multi-scale representation of the SSIM
FSIM	PIQ	A weighted combination of the phase congruency feature and the gradient magnitude feature
MS-GMSD	PIQ	The multi-scale representation of GMSD, measuring standard deviation based on pixel-wise gradient magnitude similarity map
VSI	PIQ	Similarities in the gradient magnitude and the visual saliency
HaarPSI	PIQ	local similarities and the relative importance of image areas based on Haar wavelet
VIF	PIQ	Model the image source using Gaussian scale mixtures on wavelet coefficients and quantify mutual information
LPIPS	IQA	Evaluate the Euclidean distance between image patches based on feature maps
DISTS	IQA	Combination of SSIM-like structure and texture similarity measurements based on the VGG network

## Methods

### Hologram generation

**Figure 2:**
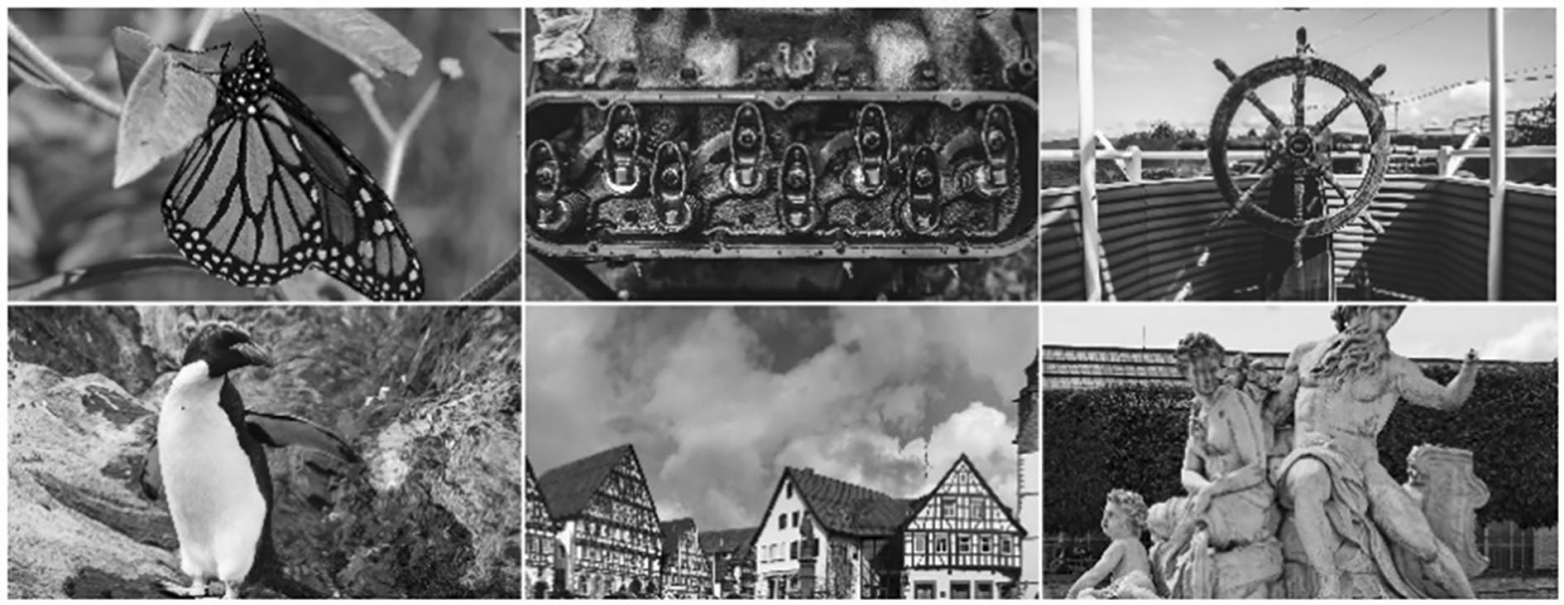
Monochrome target image amplitudes from the DIV2K dataset.

We generate CGHs for 100 high-resolution images in the DIV2K dataset^55,56^ preprocessed to give a monochrome target amplitude shown in Fig. 2. This is done for each IQM, and we therefore generate a dataset with a total of 1200 holograms. In each case we forward propagate, compare to the target, and then backward propagate to obtain the gradient for the IQM loss, which is used by the Adam optimizer to iteratively find the optimal phase hologram. In all cases we use the Adam optimizer with a 0.05 stepsize and default exponential decay rates of β1 = 0.9 and β2 = 0.999. The total number of iterations is empirically set to 1000 with the initial 15 iterations using MSE as the loss function. We apply this basic preprocessing step since initial predictions can have a significant impact on the performance of some IQMs. This step is necessary to yield acceptable optimization results and reduce the training time for learning-based IQMs. During each iteration, we normalize the amplitude of the replay field since several IQMs require input data within the range [0, 1].

The CGH generation is done on a machine with an Intel i7-8700 CPU @ 3.20 GHz and a GeForce GTX 1080 GPU. PyTorch 1.9.0 and CUDA 10.2 are used to implement complex-amplitude gradient descent optimization on the GPU. Computation takes roughly 190 GPU hours to generate the 1200 holograms to assess all 12 IQMs. Training details and computational time for each IQM loss are included in the supplementary material.

### Optical reconstruction setup

**Figure 3:**
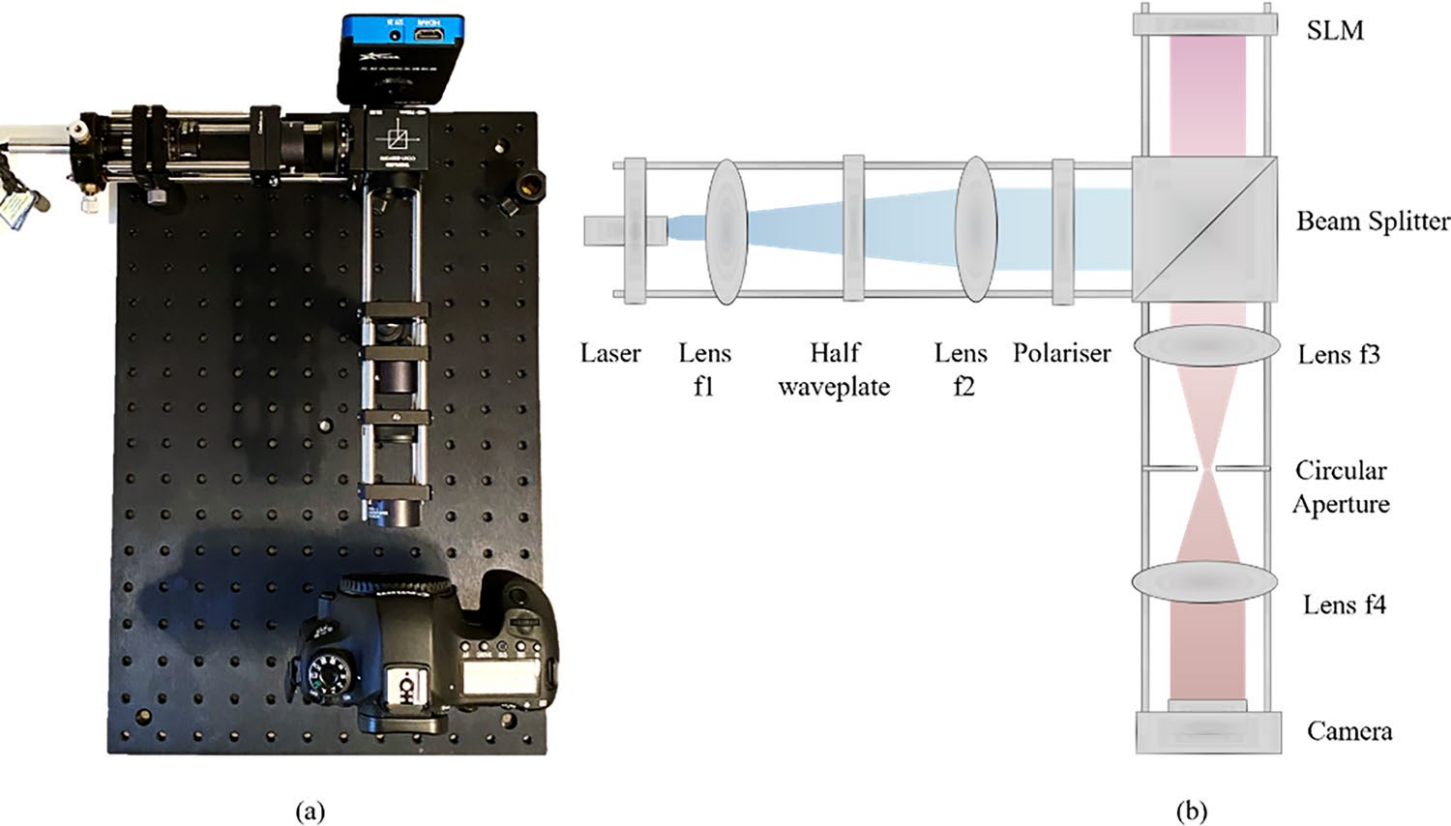
Holographic display system setup. (**a**) Our hardware display prototype with a Canon camera for image acquisition. (**b**) Optical system schematic diagram.

In order to verify our image quality from simulation, we develop a physical optical display system. We display the holograms on a SLM and optically reconstruct the replay fields captured using a camera. The proposed holographic projection system is shown in Fig. 3. Our system uses an 8-bit phase-only SLM (FSLM-2K55-P) with a pixel pitch of 6.4 µm and a resolution of 1920 × 1080. The SLM is made by the Xi’an Institute of Optics and Precision Mechanics company and is factory pre-calibrated in reflection mode. The first arm consists of a 532 nm laser source (Thorlabs CPS532), a half waveplate, a 4F lens system, and a polarizer. The 4F lens system comprises two lenses (lens 1 and lens 2) with focal lengths of 13 mm and 75 mm respectively, used to expand the beam. The expanded beam is then linearly polarized and illuminates the SLM. The second arm comprises a beam splitter and a 4F lens system with a spatial filter to reduce the DC component of the replay field and other unwanted higher diffraction orders. The focal lengths of these two lenses (lens 3 and lens 4) are 30 mm and 50 mm. The second arm is adjusted to project the reconstructed images onto the camera sensor. A neutral density filter can be inserted in the second arm to reduce the replay field intensity.

Reconstructed images are captured using a Canon EOS 6D camera without a camera lens attached. The camera output resolution is 5472 by 3648 with a gain setting of ISO 125 to minimize amplifier noise. For a fair comparison, we perform a camera calibration using a reconstructed circle grid pattern hologram and adjust the mean of captured image amplitude values to match the target image amplitude values. The target images are cropped to 1680 × 960 pixels to match the experimentally captured images. All reconstructed images are averaged out across three captured images which are captured in sRGB, the camera’s native color space. We further applied the image linearization process that converts the captured image from sRGB intensity into monochromatic linear space amplitude^13,57^.

### Subjective testing

**Figure 4:**
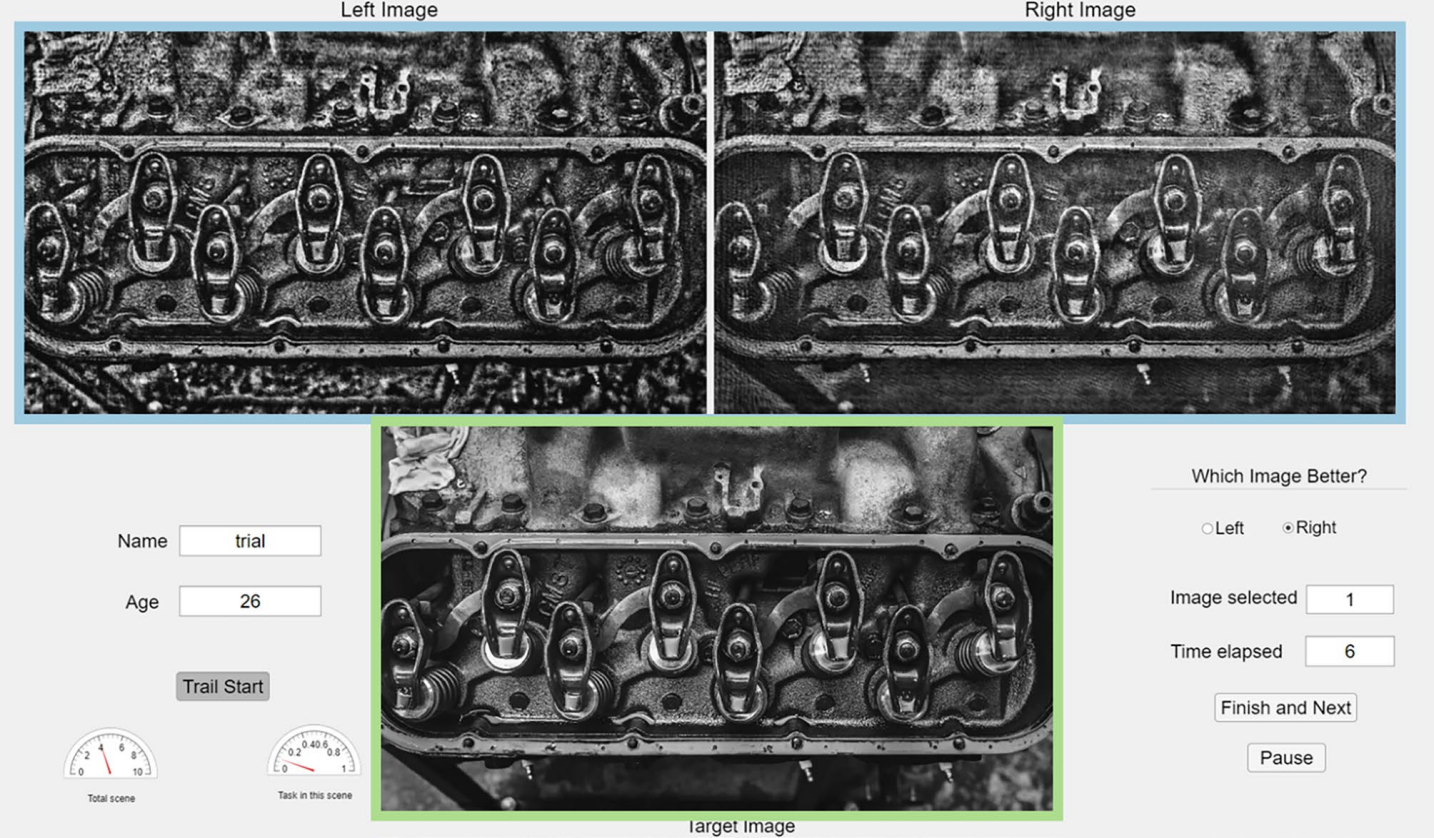
The user interface for collecting human judgments on IQM-based CGH optimization. The experimentally captured image pair from two IQM losses and the corresponding reference image are shown in the blue and the green box respectively.

To subjectively differentiate quality variations of tested models, we gather human perceptual judgments by employing a 2-alternative forced choice (2AFC) method. The experiment asks subjects to indicate which one of two distorted images is perceptually closer to the reference image. Figure 4 illustrates the interface for this experiment: an image triplet with a pair of experimentally captured images and the corresponding reference image are simultaneously presented. Subjects are asked to select the better image between two distorted ones. After the selection, two new experimentally captured images, optimized according to different IQM losses, appear on the upper screen in randomized left–right order. Progress is indicated and a pause function is available to reduce visual fatigue. The screen has a resolution of 1920 × 1080 pixels, with the displayed image resolution at $875\times 500$. The user interface supports a zoom function for careful inspection of image details.

Participants are mainly university students and are provided with appropriate instructions, including an explanation of the experimental procedure as well as a demonstration session. To avoid fatigue, we pause the user interface every 15 min and allow subjects to take a break at any time during the experiment. Experiments are performed at a normal indoor light level with reasonable varying ambient conditions according to the recommendations of ITU-R BT 500^58^. This subjective experiment was approved by the Cambridge Engineering Research Ethics committee and carried out according to the Declaration of Helsinki. We obtained informed consent and gathered paired comparisons from 20 subjects. Each subject responded to all possible combinations of generated images for a pair of target images, doing so for ten pairs of target images, yielding $\left(\genfrac{}{}{0pt}{}{12}{2}\right)\times 10=660$ stimuli. Data including time spent for each judgment, the paired-image display order and results of pairwise comparisons, is saved for analysis. The preferred image of the displayed pair contributes one point to the score of its IQM loss. Therefore, for the selected 10 sample images, each paired comparison could receive 0 to 10 points as the subjective score from the subject. In order to exclude abnormal results, we check several sentinels in each observation data that consist of pairs with obvious visual quality contrast. Overall, we received 13,200 judgments across 12 IQM losses, and each loss is ranked 1100 times. The average time for one judgment is approximately 3 s.

We employ the Bradley-Terry model^59,60^ to aggregate pairwise comparisons and obtain a global ranking of IQM losses for CGH optimization based on the subjective data. From partial orderings provided in the data, we wish to infer not only the ranking order of tested losses but also the subjective visual quality scores associated with the losses themselves. If we denote $s=[{s}_{1},{s}_{2},{s}_{3},\dots {s}_{m}]$ as subjective scores of the evaluated IQM losses, the Bradley-Terry model assumes that the probability of choosing loss $i$ over loss $j$ is:

6 $${p}_{ij}=\frac{{e}^{{s}_{i}}}{{e}^{{s}_{i}}+{e}^{{s}_{j}}}.$$

Given the observed number of times that IQM loss $i$ is favored over IQM loss $j$ as ${w}_{ij}$, We then can obtain the likelihood of $i$ over $j$ as ${p}_{ij}^{{w}_{ij}}$.Thus, assuming outcomes of each paired comparison are statistically independent, the likelihood function of all $(i,j)$ pairs is defined by:

7 $$P=\prod_{i=1}^{M} \prod_{\genfrac{}{}{0pt}{}{j=1}{j\ne i}}^{M} {p}_{ij}^{{w}_{ij}}.$$

The subjective score for IQM loss ${s}_{i}$ can then be jointly estimated by maximizing the log-likelihood of all pairwise comparison observations:

8 $$\ell \left({s}_{i}\right)=\sum_{i=1}^{M} \sum_{\genfrac{}{}{0pt}{}{j=1}{j\ne i}}^{M} \left({w}_{ij}{s}_{i}-{w}_{ij}\mathrm{log}\left({e}^{{s}_{i}}+{e}^{{s}_{j}}\right)\right).$$

## Results and discussion

### Hologram generation results

**Figure 5:**
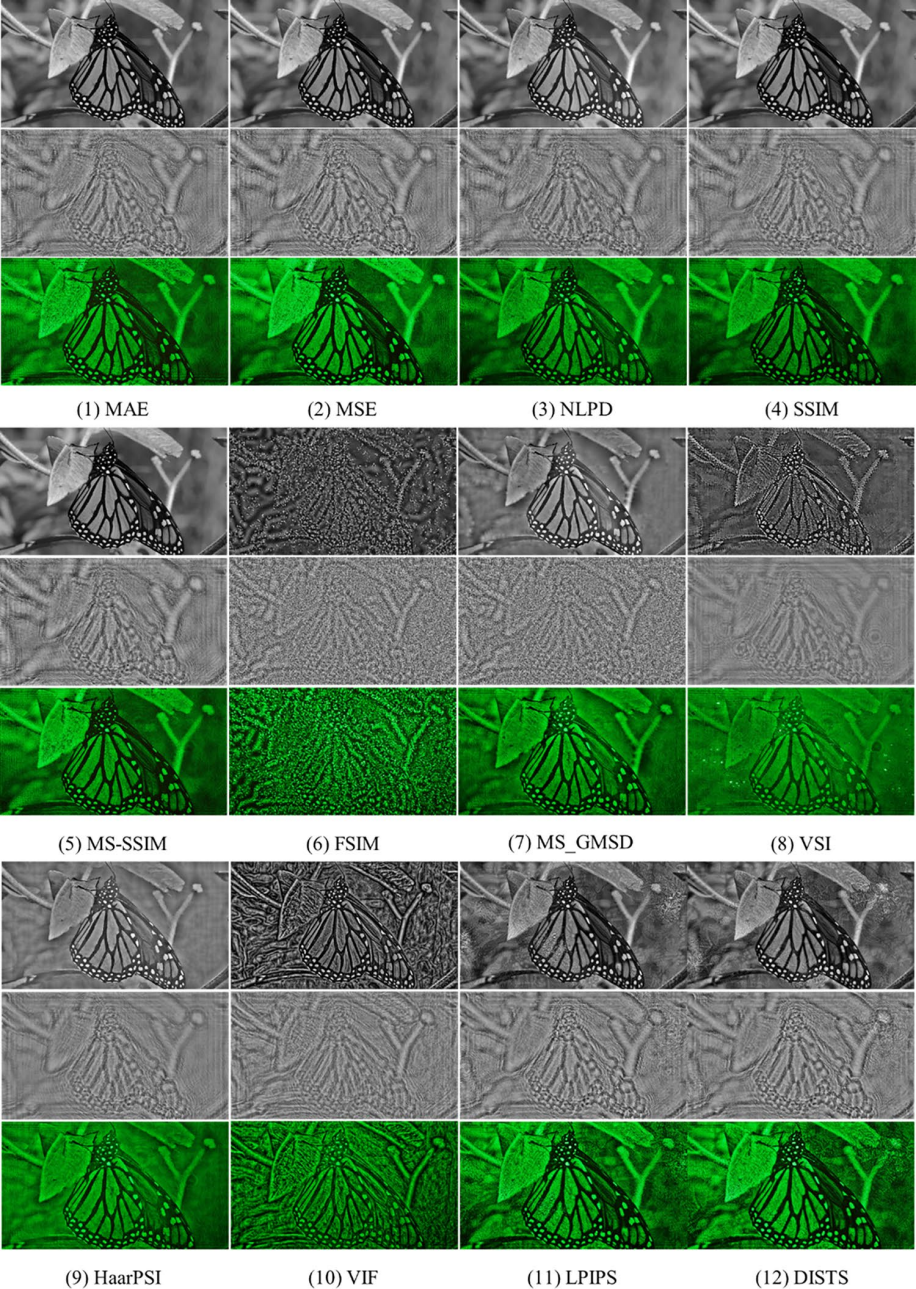
Simulated and captured results for CGH optimization using twelve different IQM losses. We show the reconstructed image at the top for each loss with the phase hologram in the middle and its corresponding captured results at the bottom.

The simulated reconstruction results based on IQM optimization models are shown in Fig. 5. Corresponding phase holograms, as well as the experimental captured results in sRGB space, are shown in the second and third rows respectively.

### Qualitative interpretation

**Figure 6:**
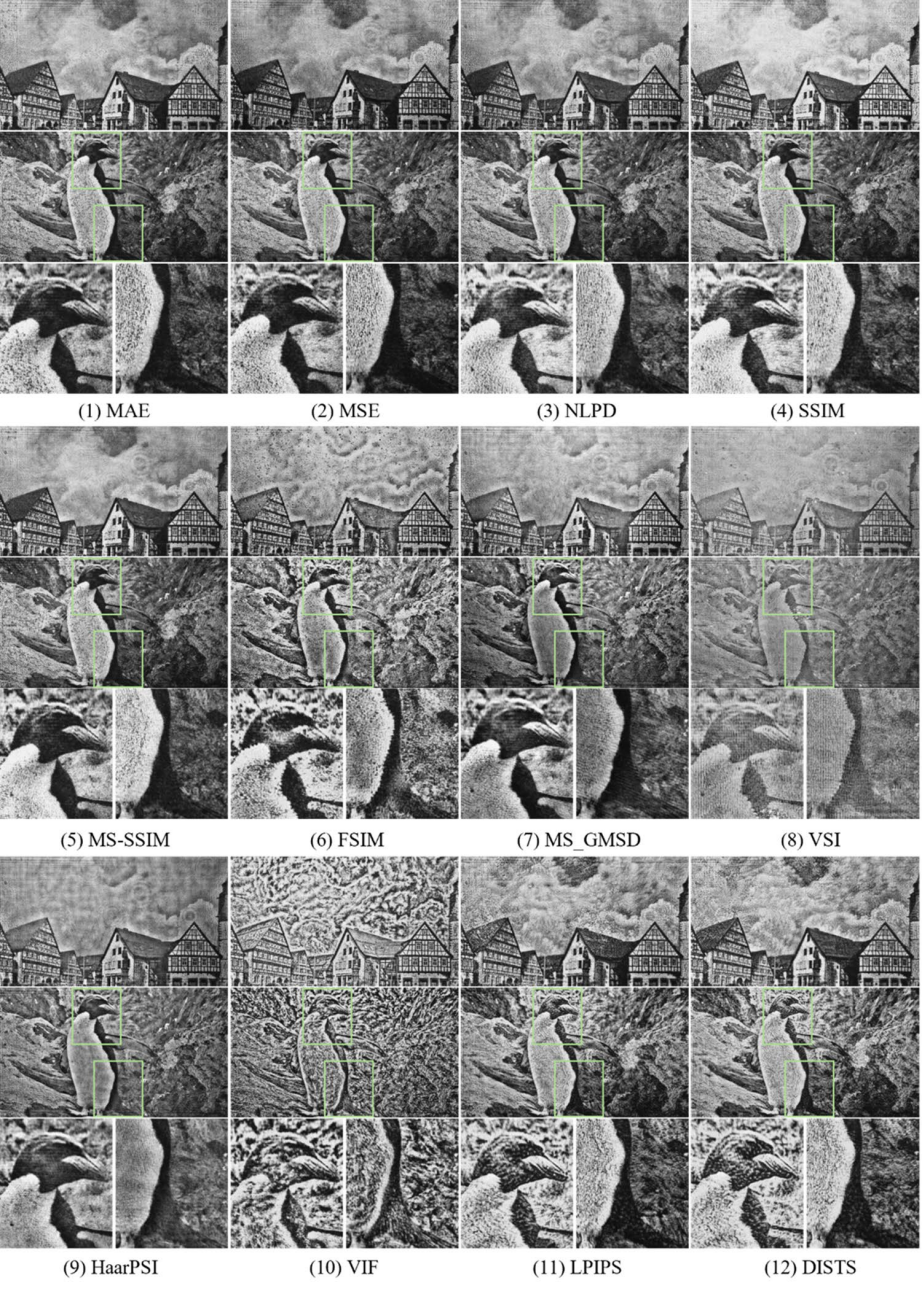
Captured reconstruction results. For target images, we display phase holograms optimized by IQM losses. Reconstructions results of IQM losses are captured with our holographic display prototype for image quality comparison.

We first make a qualitative comparison across all IQM-optimized methods for experimental results. As shown in Fig. 6, most IQM-based optimization models converge on a reasonable visual quality. We observe that MAE, MSE, NLPD, SSIM, and MS-SSIM perform well but have undesirable local noise, which can be observed in the image patches selected from the reconstructed images. FSIM and VIF amplify high-frequency information, leading to structural over-enhancement. VSI, MS-GMSD and HaarPSI preserve the overall structures with a smooth appearance, but artificially reduce local contrast with noticeable artifacts. Models based on deep-learning methods such as LPIPS and DISTS can recover the target image details but superimpose textures on the image.

The optically reconstructed images exhibit laser speckle noise and are subject to optical aberrations, resulting in some noticeable common artifacts across all IQMs, including ghost and ripple effects. The dynamic range of the camera is limited and captured images are prone to photometric distortions, including reduced contrast and saturation.

### Objective interpretation

We use the proposed IQMs as quality measures to evaluate the performance of gradient descent based CGH optimization using different IQM losses. All IQMs are used to objectively evaluate the captured results. Scores are averaged over all 100 images for each metric and for each IQM-based loss shown in Table 2. Each element indicates the score of an IQM loss evaluated using another IQM as a quality predictor.

**Table 2:** Objective performance of IQM-based model evaluated by IQMs as quality metrics.

IQM losses	Objective image quality metrics											
	MAE	MSE	NLPD	SSIM	MS-SSIM	FSIM	MS-GMSD	VSI	HaarPSI	VIF	LPIPS	DISTS
MAE	0.104	0.021	0.754	0.382	0.568	0.770	0.270	0.893	0.264	0.118	0.600	0.265
MSE	0.120	0.028	0.862	0.315	0.458	0.720	0.276	0.877	0.234	0.078	0.618	0.273
NLPD	0.118	0.024	0.717	0.365	0.566	0.783	0.258	0.905	0.287	0.117	0.601	0.271
SSIM	0.107	0.021	0.739	0.371	0.563	0.779	0.262	0.904	0.279	0.112	0.604	0.272
MS-SSIM	**0.096**	**0.018**	**0.696**	**0.414**	**0.610**	**0.795**	0.256	**0.913**	0.296	**0.133**	0.589	0.253
FSIM	0.185	0.058	1.083	0.219	0.305	0.648	0.294	0.795	0.187	0.067	0.664	0.387
MS-GMSD	0.153	0.040	0.833	0.328	0.451	0.744	0.258	0.879	0.274	0.098	0.608	0.283
VSI	0.158	0.040	0.816	0.299	0.430	0.761	0.256	0.894	0.276	0.079	0.628	0.406
HaarPSI	0.145	0.035	0.748	0.380	0.526	0.783	**0.245**	0.901	**0.313**	0.121	0.589	0.272
VIF	0.171	0.051	0.895	0.338	0.413	0.633	0.294	0.790	0.200	0.197	**0.580**	0.314
LPIPS	0.127	0.029	0.896	0.288	0.430	0.696	0.289	0.852	0.216	0.084	0.635	0.247
DISTS	0.130	0.030	0.911	0.279	0.415	0.690	0.289	0.852	0.212	0.077	0.636	**0.246**

Significant values are in bold.

By inspecting each row of the metric table, we find MAE, NLPD, SSIM, and MS-SSIM maintain the best performance among all IQM losses as previously predicted by the qualitative comparison. MS-SSIM loss produces superior reconstruction quality and objectively ranks as the best performing IQM-based CGH optimization model on most evaluation metrics, while FSIM ranks as the least preferred method. Several other IQM losses, including NLPD, MAE, SSIM, HaarPSI and MS-GMSD, also outperform the MSE loss, which objectively validates the use of IQMs for CGH optimization.

Since the PIQ library implements its own SSIM and MS-SSIM metrics for image quality assessment, we can further evaluate our top-performing models by using these metrics, as shown in Table 3. Though both the IQA and PIQ libraries have been benchmarked on a set of common databases and have nearly consistent ranking results in model evaluation, there is disagreement with the actual values of performance evaluation, with the IQM library generally obtaining lower scores. Hence, in the absence of a standard IQM implementation, it becomes more challenging to compare the performance of different algorithms.

**Table 3:** Objective performance of IQM-based model evaluated on different libraries.

IQM losses	Objective image quality metrics			
	SSIM	MS-SSIM	SSIM (piq)	MS-SSIM (piq)
MS-SSIM	0.414	0.610	0.619	0.641
NLPD	0.365	0.566	0.567	0.601
HaarPSI	0.380	0.526	0.550	0.591
MAE	0.382	0.568	0.577	0.602
SSIM	0.371	0.563	0.568	0.596
MS-GMSD	0.328	0.451	0.463	0.505
MSE	0.315	0.458	0.446	0.484

### Subjective interpretation

**Figure 7:**
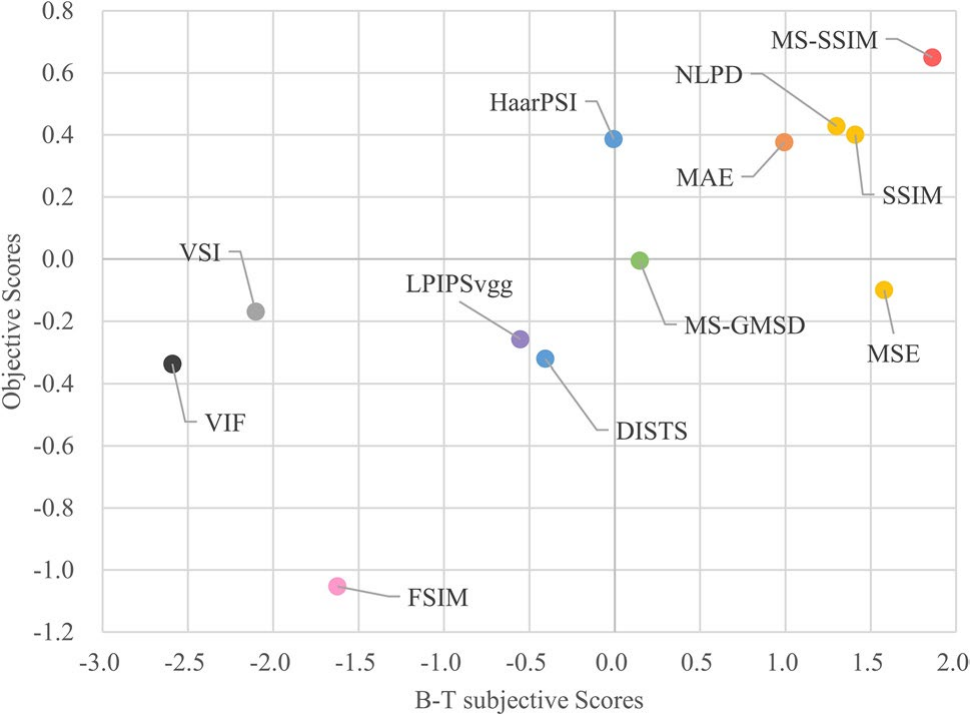
Quantitative comparison of IQM-based CGH optimization. Scatter points represent the losses for CGH optimization. Points with the same color are statistically indistinguishable for subjective results. Vertical and horizontal axes indicate the objective performance and the subjective performance of each loss respectively.

We implement the Bradley-Terry model in R to iteratively solve the given equation Eq. (8) and obtain the optimal estimate ${s}_{i}$ for each model. The Bradley-Terry model scores are normalized by shifting to zero means, resulting in a global ranking of perceptual optimization performance. We further conduct independent two-sample two-tailed t-tests to investigate whether the differences between the subjective performance of IQM losses are statistically significant. Specifically, we consider that the obtained observations from participants are normally distributed under the null hypothesis and compare the ranking scores for any of the two losses. If the comparison cannot reject the null hypothesis of no difference at the standard significance level $\alpha =0.05,$ we put the evaluated losses in the same group as they are statistically indistinguishable. Figure 7 shows the scatter plot of the combined subjective and objective performance of tested IQM losses for CGH optimization. Scatter points with the same color are in the same statistical significance group for subjective tests. The objective global ranking score for each IQM loss can be obtained by adding ranking orders from all quality metrics derived from Table 2 and normalizing them to zero mean. Scores have been reformulated to ensure a higher score indicates a higher predicted quality.

The scatter plot indicates that the MS-SSIM is the top-ranking loss function, as agreed upon by both subjective and objective evaluations. NLPD and SSIM losses are statistically indistinguishable from the MSE loss for subjective performance. The MSE loss unexpectedly achieves higher performance in the subjective test than HaarPSI, and MAE losses, despite performing far worse in objective performance. A similar trend also occurs in VSI and VIF losses versus FSIM loss. This disagreement is due to different objective and subjective weighting strategies on image structure similarity, image smoothness, luminance, and contrast.

We further calculate Spearman’s rank-order correlation coefficient (SRCC) between objective and subjective scores, as shown in Table 4. Higher SRCC scores indicate a better correlation of a metric with subjective ratings. Although most modern image quality metrics show superior performance in existing image databases, we observe that for CGH they have less correlation than pixel-error-based metrics to human judgments. This may be because the most common image databases for benchmarking such LIVE^61^, TID2008^62^ and TID2013^63^ comprise source images with synthetically distorted images. The synthetic distortion types, including White Gaussian Noise, JPEG2000 compression, and Gaussian Blur with varied distortion levels, attempt to reflect various image impairments found in image processing. Experimental CGH reconstructed images, such as those seen here, can be rather more complex with more types of distortions produced during the optical reconstruction and image acquisition. Furthermore, CGHs are predominantly tainted by noise, whereas some IQMs were developed for recognizing blurry objects, inferring details in deblurred objects, or super-resolution imaging tasks. Current IQMs are not well specifically benchmarked for those real-world and CGH distortions. For partial coherent light illumination in the holographic optical system that could bring more blurry effect and contrast reduction in the replay field^57,64^, modern IQMs may take advantage in inferring blurry and contrast-reduced information. Therefore, the use of IQMs may potentially have better performance in partial coherent holographic displays.

**Table 4:** SRCC between objective scores and subjective scores of IQM-based CGH optimization.

Objective image quality metrics	SRCC
MAE	0.846
MSE	0.825
NLPD	0.657
SSIM	0.587
MS-SSIM	0.839
FSIM	0.692
MS-GMSD	0.434
VSI	0.678
HaarPSI	0.566
VIF	0.189
LPIPS	0.266
DISTS	0.427

## Conclusion

In this work, we have conducted a comprehensive study of the real-world performance of using IQM as loss functions in the CGH optimization process. By benchmarking with a standard optical reconstruction dataset, we have collated the results of applying 12 distinct IQMs as loss functions in both objective and subjective ratings. The results from the comparison study show that IQM losses can achieve better image quality than the MSE loss in generating holograms, with the MS-SSIM loss outperforming all the other losses. This extensive comparison provides guidance for finding a specific perceptually-motivated loss function for CGH generation.

Beyond this study, individual IQM losses can be further combined based on their complementarity to incorporate the specific CGH distortions. We recognize that our analysis is limited to 2D hologram reconstruction. For 3D holographic applications, the authors believe that there are several extensions to the work conducted in this study, such as the use of blurring distortion, which could be a significant perceptual factor to be considered in hologram optimization.

## Supplementary Information

Supplementary Information 1.

Supplementary Information 2.

## Data Availability

The datasets generated and/or analysed during the current study are available in the GitHub repository, https://github.com/fy255/perceptual_cgh.

## Code availability

The code for hologram generation and evaluation are publicly available in the GitHub repository, https://github.com/fy255/perceptual_cgh, Additional codes are available from the corresponding authors upon reasonable request.
