# Supplementary material for: Perceptually motivated loss functions for computer generated holographic displays
Source: Sci Rep. 2022 May 11;12:7709. doi: 10.1038/s41598-022-11373-8 (PMC9095705; doi:10.1038/s41598-022-11373-8)
Supplement: Supplementary file 2 — Supplementary Information 2. [file 41598_2022_11373_MOESM2_ESM.docx]

Perceptually Motivated Loss Functions for Computer Generated Holographic Displays: Supplementary information

Fan Yang ^1, 2^, Andrew Kadis ^1^, Ralf Mouthaan ^1^, Benjamin Wetherfield ^1^, Andrzej Kaczorowski ^2^, and Timothy D. Wilkinson ^1, *^

^1^ Centre of Molecular Materials, Photonics and Electronics, University of Cambridge, Cambridge, United Kingdom

^2^ VividQ Ltd., Research Division, Cambridge, UK

^*^ Corresponding author: tdw13@cam.ac.uk

S1: Objective ranking results of IQM-based model evaluated by IQMs as quality metrics

We converted the Table 2 into a 2D ranking plot to give a well-defined and more illustrative comparison. Horizontal axis indicates IQMs as quality measure used to evaluate the objective performance, and vertical axis indicates IQMs used as loss functions for CGH optimization. The rank order is colour coded from green to red with numbers of 1–12 to indicate performance from best to the worst.


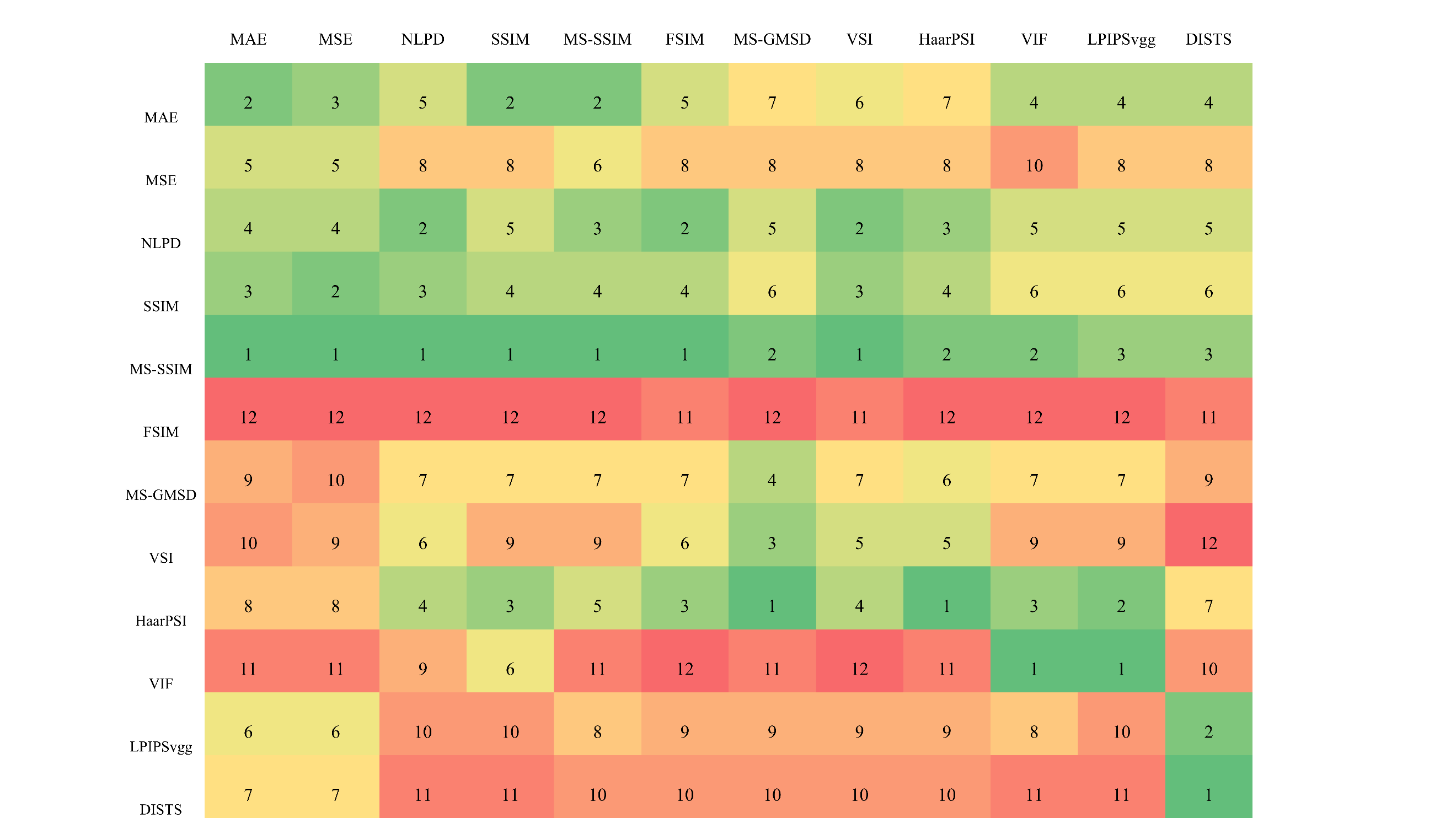


Fig. S1. Objective ranking plot of IQM-based model evaluated by IQMs as quality metrics

S2: Subjective ranking results from participants

We converted the pairwise comparisons of generated images from each subject using the B-T model to obtain the ranking order of IQM losses shown in each column in Table S1. Numbers 1 to 12 denote the rank order from the best to the worst.

Table S2 shows the winning matrix voted by all participants. Each element in the table indicates the number of votes that the column method is preferred to the row method. We received overall 13200 judgments across 12 IQM losses.

Table S3 indicates the B-T scores calculated from the winning matrix. We further conducted independent two-sample t-tests with two-tailed distribution to investigate whether the adjacent methods in the B-T ranking are statistically distinguishable.

Table S1. Subjective ranking results from participants. Each column indicates the ranking of IQM losses evaluated by a subject. Numbers 1 to 12 denote the rank order from the best to the worst.

| subjects | MAE | MSE | NLPD | SSIM | MS-SSIM | FSIM | MS-GMSD | VSI | HaarPSI | VIF | LPIPS | DISTS |
| --- | --- | --- | --- | --- | --- | --- | --- | --- | --- | --- | --- | --- |
| 1 | 5 | 4 | 2 | 3 | 1 | 10 | 6 | 11 | 7 | 12 | 9 | 8 |
| 2 | 5 | 2 | 4 | 3 | 1 | 10 | 6 | 11 | 8 | 12 | 9 | 7 |
| 3 | 5 | 2 | 4 | 3 | 1 | 10 | 8 | 11 | 9 | 12 | 7 | 6 |
| 4 | 6 | 2 | 4 | 3 | 1 | 10 | 8.5 | 12 | 7 | 11 | 8.5 | 5 |
| 5 | 7 | 4 | 3 | 1 | 2 | 10.5 | 5 | 10.5 | 6 | 12 | 9 | 8 |
| 6 | 5 | 2 | 3 | 4 | 1 | 11 | 6.5 | 10 | 6.5 | 12 | 8 | 9 |
| 7 | 6 | 4 | 1 | 2 | 3 | 11 | 5 | 10 | 7 | 12 | 9 | 8 |
| 8 | 5 | 3 | 4 | 2 | 1 | 9 | 6 | 10 | 7 | 12 | 8 | 11 |
| 9 | 2 | 1 | 3.5 | 5 | 3.5 | 10 | 6 | 11 | 7 | 12 | 9 | 8 |
| 10 | 5 | 2 | 4 | 3 | 1 | 10 | 6 | 11 | 7 | 12 | 9 | 8 |
| 11 | 2 | 1 | 5 | 4 | 3 | 11 | 6 | 12 | 7.5 | 10 | 9 | 7.5 |
| 12 | 5 | 1 | 4 | 2 | 3 | 10 | 6.5 | 11 | 8 | 12 | 9 | 6.5 |
| 13 | 5 | 2 | 4 | 3 | 1 | 10 | 7.5 | 11 | 6 | 12 | 7.5 | 9 |
| 14 | 5 | 1 | 3.5 | 3.5 | 2 | 10 | 6 | 11 | 7 | 12 | 9 | 8 |
| 15 | 3 | 2 | 5 | 4 | 1 | 10 | 6 | 12 | 8 | 11 | 7 | 9 |
| 16 | 5 | 4 | 2.5 | 2.5 | 1 | 10 | 6 | 12 | 7 | 11 | 9 | 8 |
| 17 | 3 | 2 | 4.5 | 4.5 | 1 | 10 | 6.5 | 11 | 9 | 12 | 8 | 6.5 |
| 18 | 5 | 4 | 3 | 1 | 2 | 10 | 8 | 11 | 6 | 12 | 9 | 7 |
| 19 | 4 | 3 | 5 | 1 | 2 | 10 | 6 | 11 | 7 | 12 | 9 | 8 |
| 20 | 5 | 2 | 3 | 4 | 1 | 10 | 6 | 11 | 7 | 12 | 9 | 8 |

Table S2. Subjective winning matrix voted by all participants.

|  | MAE | MSE | NLPD | SSIM | MS-SSIM | FSIM | MS-GMSD | VSI | HaarPSI | VIF | LPIPS | DISTS |
| --- | --- | --- | --- | --- | --- | --- | --- | --- | --- | --- | --- | --- |
| MAE | 0 | 78 | 104 | 81 | 53 | 178 | 138 | 190 | 131 | 197 | 167 | 162 |
| MSE | 122 | 0 | 124 | 118 | 71 | 197 | 152 | 198 | 171 | 194 | 174 | 182 |
| NLPD | 96 | 76 | 0 | 91 | 93 | 196 | 167 | 200 | 156 | 193 | 168 | 158 |
| SSIM | 119 | 82 | 109 | 0 | 85 | 189 | 161 | 196 | 173 | 193 | 177 | 162 |
| MS-SSIM | 147 | 129 | 107 | 115 | 0 | 194 | 170 | 199 | 174 | 197 | 190 | 178 |
| FSIM | 22 | 3 | 4 | 11 | 6 | 0 | 25 | 115 | 35 | 132 | 63 | 58 |
| MS-GMSD | 62 | 48 | 33 | 39 | 30 | 175 | 0 | 198 | 105 | 183 | 132 | 124 |
| VSI | 10 | 2 | 0 | 4 | 1 | 85 | 2 | 0 | 8 | 158 | 25 | 42 |
| HaarPSI | 69 | 29 | 44 | 27 | 26 | 165 | 95 | 192 | 0 | 186 | 120 | 113 |
| VIF | 3 | 6 | 7 | 7 | 3 | 68 | 17 | 42 | 14 | 0 | 25 | 27 |
| LPIPS | 33 | 26 | 32 | 23 | 10 | 137 | 68 | 175 | 80 | 175 | 0 | 89 |
| DISTS | 38 | 18 | 42 | 38 | 22 | 142 | 76 | 158 | 87 | 173 | 111 | 0 |

Table S3. Bradley-Terry scores and p-values of the t-test by comparing adjacent methods.

|  | MS-SSIM | MSE | SSIM | NLPD | MAE | MS-GMSD | HaarPSI | | DISTS | LPIPS | FSIM | VSI | VIF |
| --- | --- | --- | --- | --- | --- | --- | --- | --- | --- | --- | --- | --- | --- |
| B-T scores | 1.861 | 1.578 | 1.409 | 1.298 | 0.993 | 0.146 | | -0.007 | -0.407 | -0.553 | -1.625 | -2.103 | -2.591 |
| P-value (adjacent) | N/A | 1.787e-02 | 1.368e-01 | 7.909e-02 | 4.900e-03 | 1.782e-05 | | 5.714e-03 | 1.009e-01 | 2.227e-02 | 3.521e-10 | 6.100e-06 | 3.561e-04 |

S3: Additional captured images


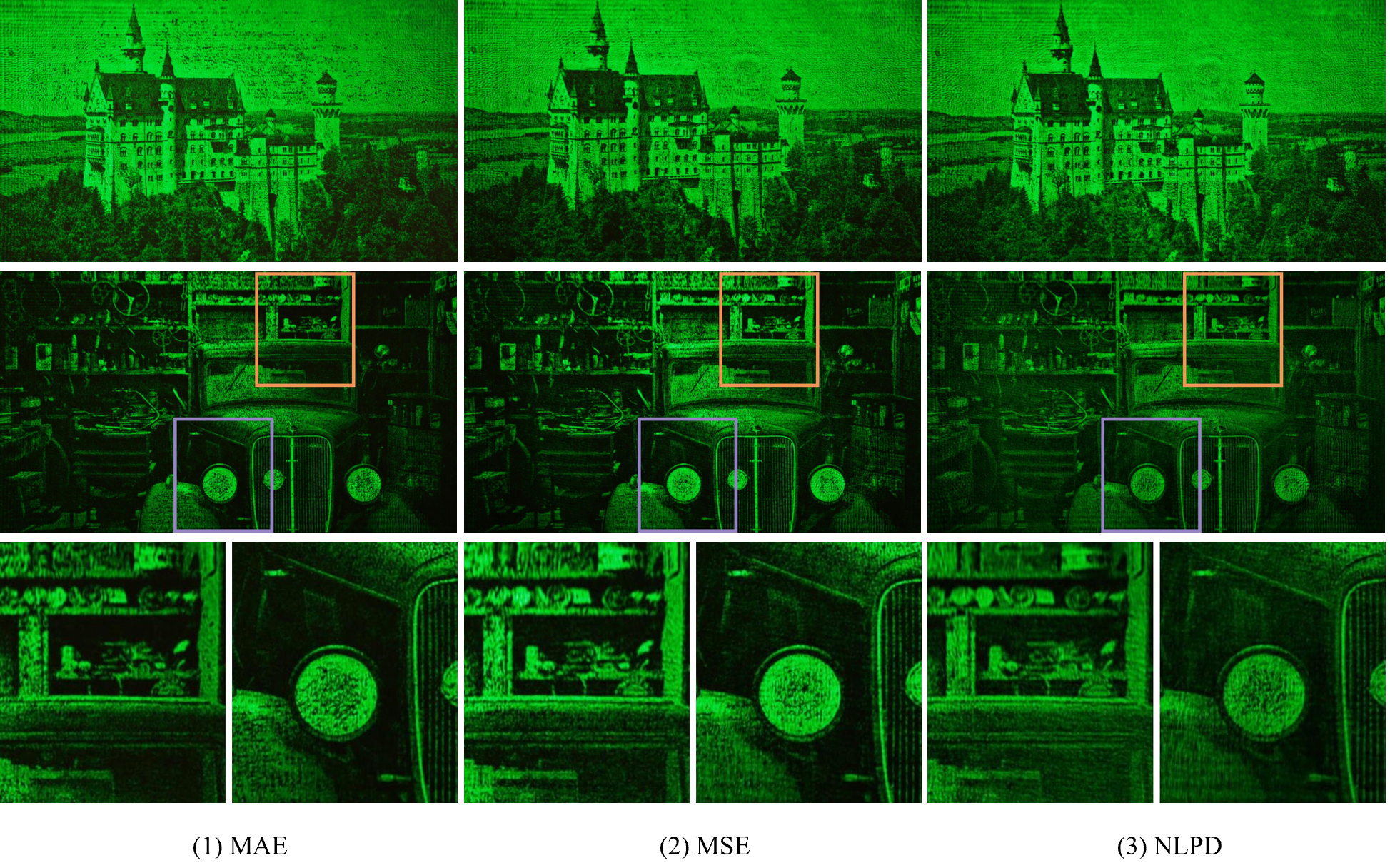


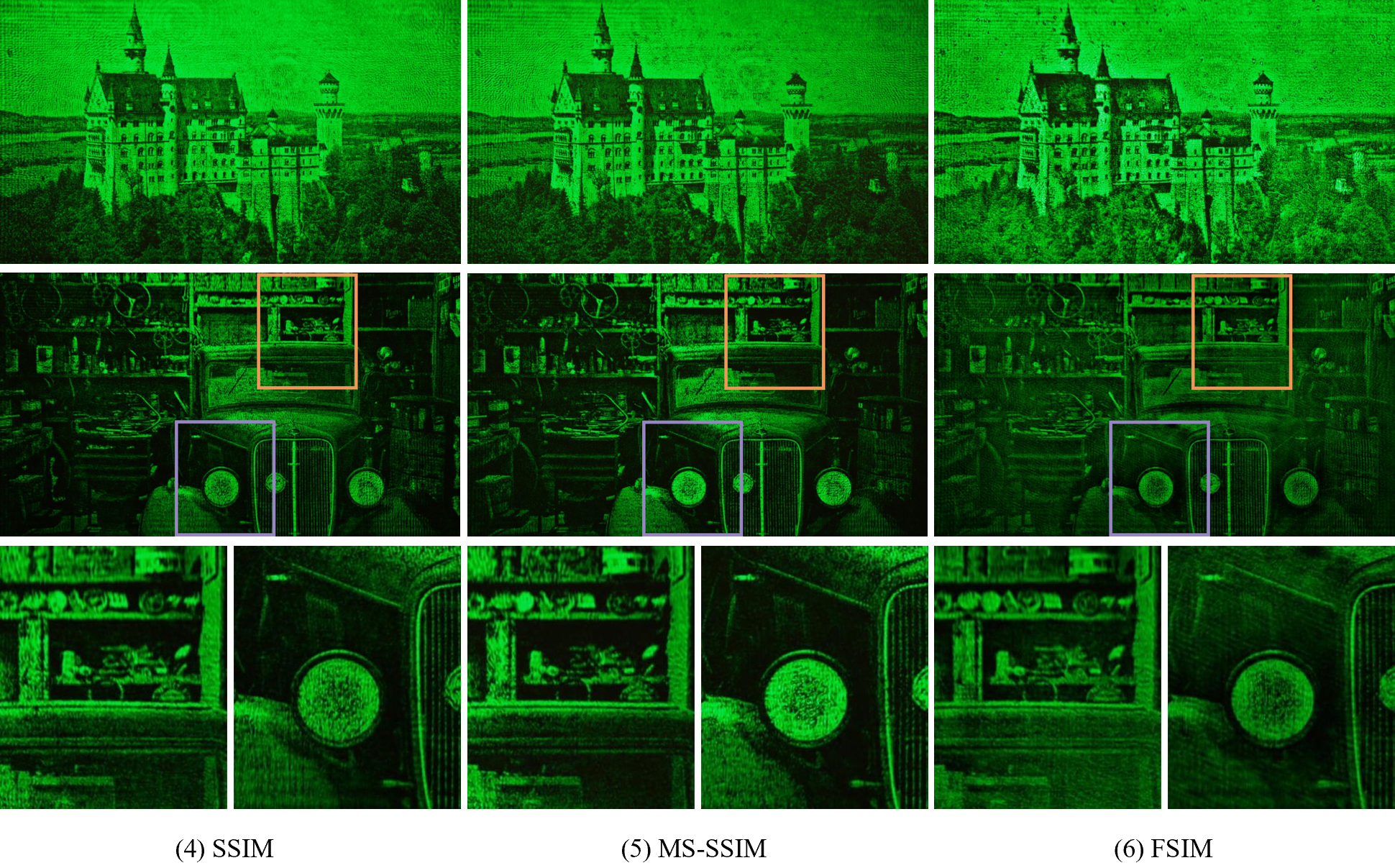


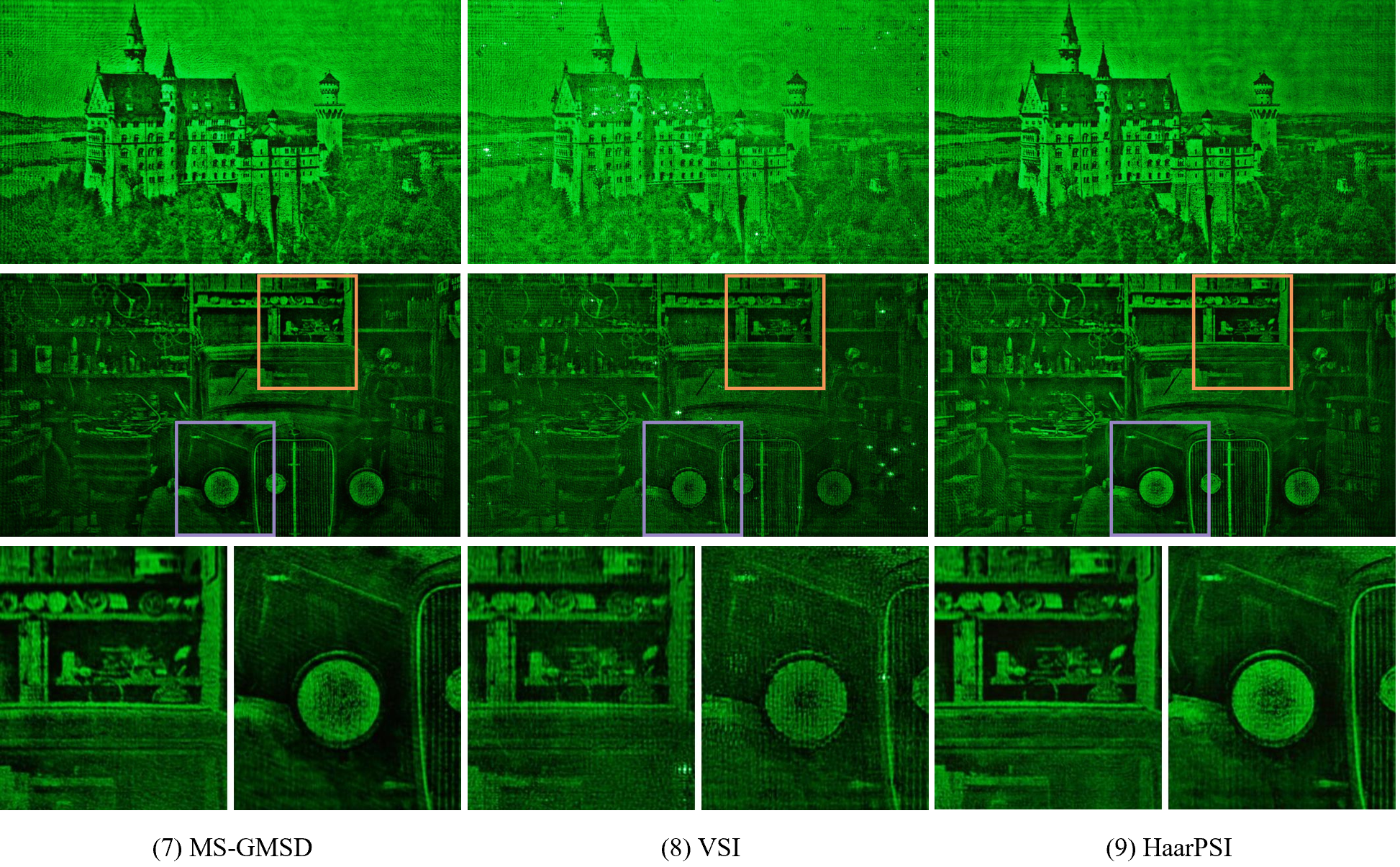


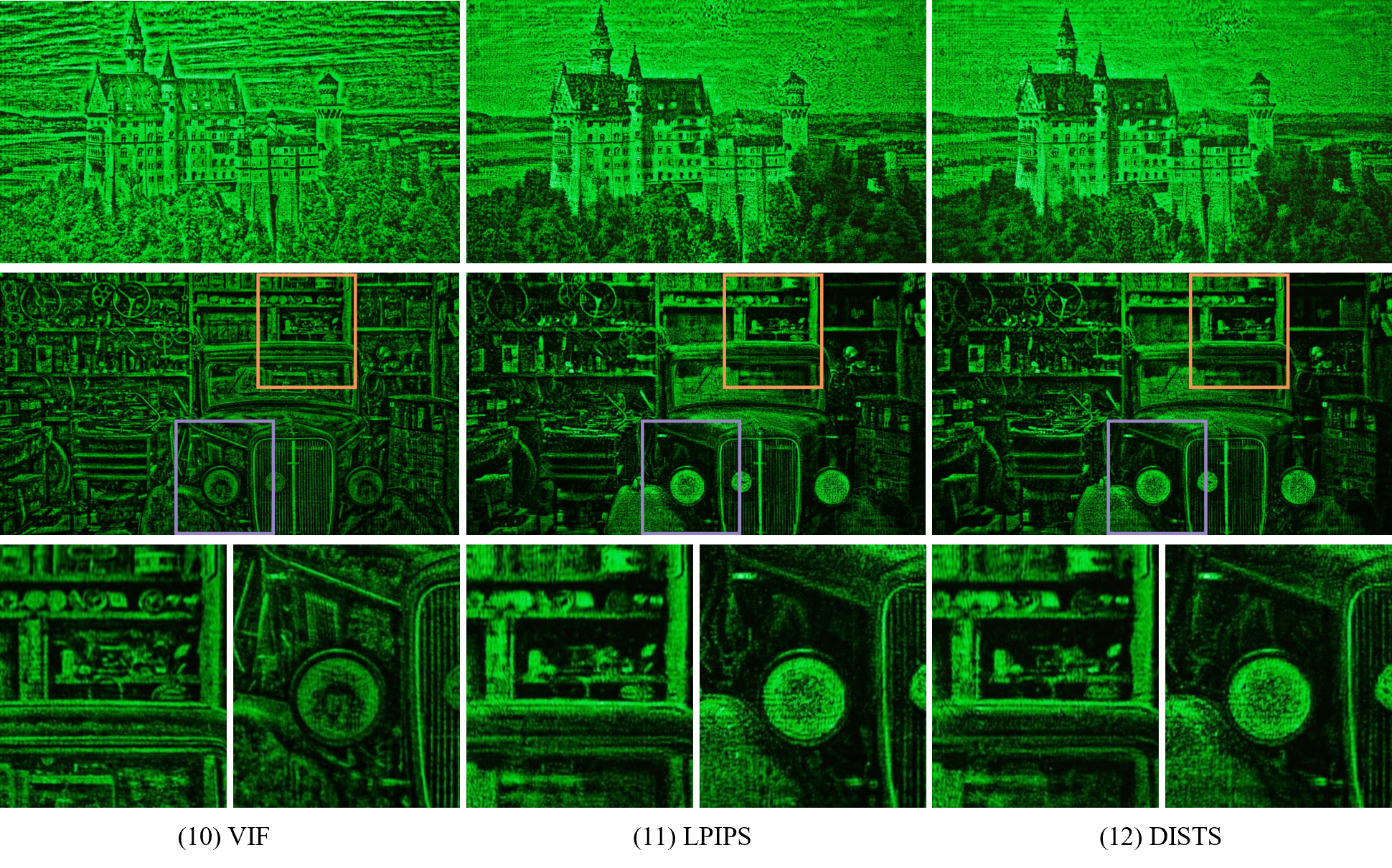


Fig. S2. Additional captured reconstruction results for the top six IQM losses with zoom-in details. We display phase holograms optimized by IQM losses. Reconstructions results of IQM losses are captured with our holographic display prototype for image quality comparison.

S4: Training details and computational time for each IQM loss

Table S4. Computational time for each IQM loss

|  | MAE | | MSE | NLPD | SSIM | MS-SSIM | FSIM | MS-GMSD | VSI | HaarPSI | VIF | LPIPS | DISTS |
| --- | --- | --- | --- | --- | --- | --- | --- | --- | --- | --- | --- | --- | --- |
| Time per image(min.) | | 1.5 | 1.78 | 3.72 | 2.50 | 2.88 | 2.52 | 2.48 | 2.58 | 2.40 | 21.30 | 33.33 | 33.33 |
| Total time (hr.) | | 2.50 | 2.97 | 6.20 | 4.17 | 4.80 | 4.20 | 4.13 | 4.30 | 4.00 | 35.50 | 55.56 | 55.56 |


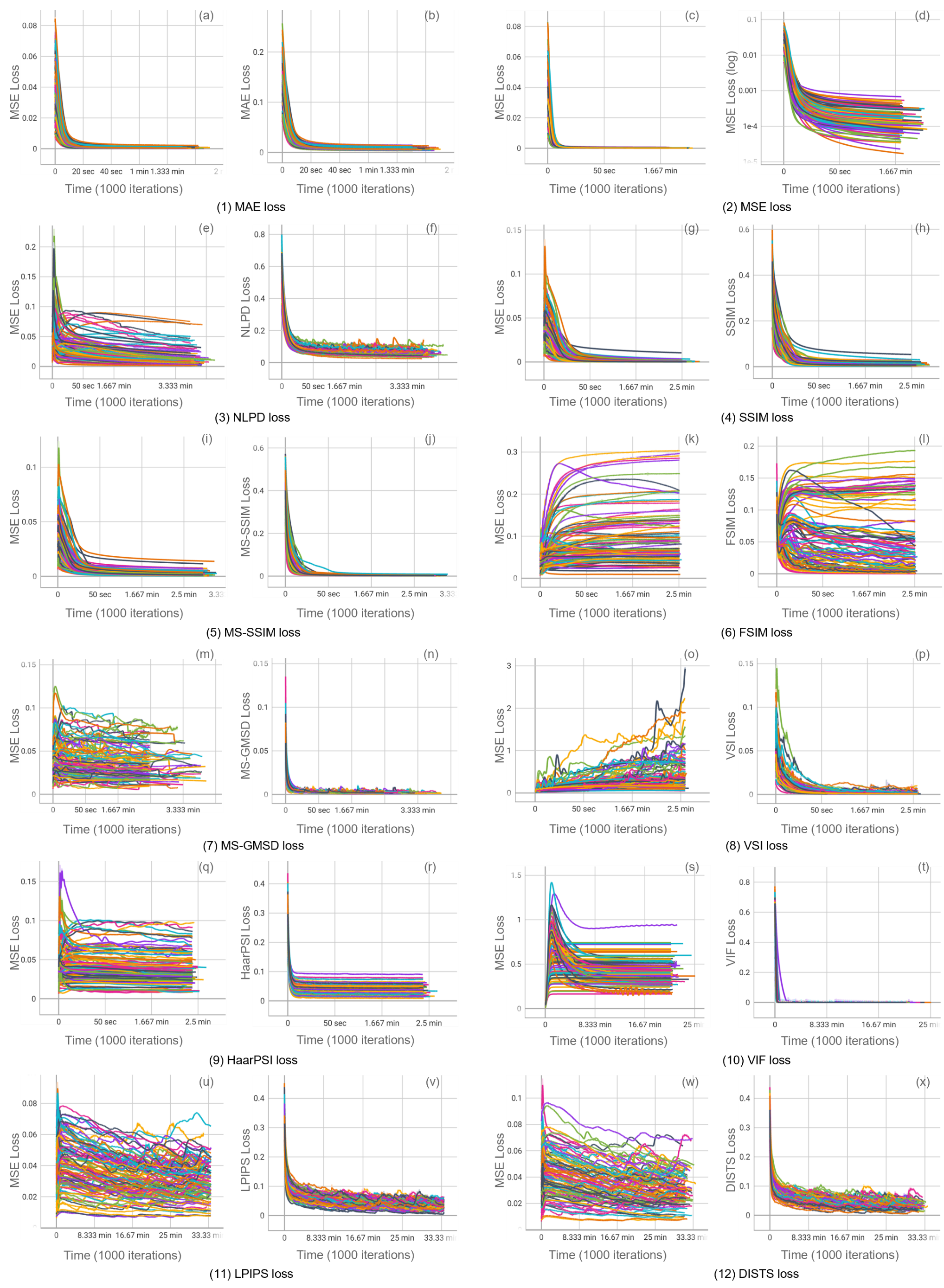


Fig. S3. Computational time of each image and the training details of IQM losses for CGH optimization. We plot all runs of images for each IQM loss function, showing how the MSE loss and its own metric loss change with each iteration.

S5: Challenges on holographic subjective experiment

In order to verify our image quality from simulation, we introduce a camera to first record then display the reconstructed images for subjective experiment. Comparing with the alternative option that directly displaying the experimental reconstructed images on a screen for human perceptual judgments, this experimental choice may suffer from distortions produced during image acquisition and the replay process, which could adversely affect subjective evaluations on the reconstructed image quality. However, since all IQM methods are going through the same experimental procedure and suffer from the same distortions, this experimental choice should have the same effect for all methods and have little impact for IQM method comparison. As pointed out in references^1–3^, establishing the subjective evaluation on experimental CGHs is quite challenging. First, there is no widely accepted testing methodology for subjectively evaluating the CGH reconstructed image quality. A common practice for subjective evaluation is to numerically reconstruct the CGH and display the reconstruction on high-end 2D monitors. Second, there is no widely accepted configuration of high-end holographic displays for subjective pair comparison benchmarking. Most holographic displays are operated under customized laboratory conditions with no standard procedure for calibrating, characterizing, and testing holographic displays. Moreover, holographic displays suffer from practical issues including limited FoV, overall size, eyebox, laser speckles, eye safety issue with laser illumination, and optically alignment for the same testing condition per test subject etc. Those practical issues add another layer of complexity for holographic data benchmarking. Finally, for a fair comparison, both objective and subjective assessment of CGH data should be taken under the same visual condition. A direct holographic projection for subjective evaluation without recording cannot be evaluated by objective quality metrics.

Reference

1. Kizhakkumkara, R. *et al.* PEG Pleno Holography Common Test Conditions V1.0. in *WG1N88040, 88th JPEG Meeting* (2020).
2. Ahar, A. *et al.* Comprehensive performance analysis of objective quality metrics for digital holography. *Signal Processing: Image Communication* 97, 116361 (2021).
3. Ahar, A. *et al.* Suitability analysis of holographic vs light field and 2D displays for subjective quality assessment of Fourier holograms. *Optics Express* 28, 37069–37091 (2020).
